# Predicting early clinical success of prostate artery embolization using MRI perfusion in benign prostatic hyperplasia

**DOI:** 10.1186/s42155-025-00562-x

**Published:** 2025-05-21

**Authors:** Skander Sammoud, Fabien De Oliviera, Tarek Kammoun, Houssem Loukil, Ghizlane Touimi Ben Jelloun, Sinda Ghabri, Wissem Nabi, Stéphane Droupy, Pascal Gres, Jean-Paul Beregi, Julien Frandon

**Affiliations:** 1 IMAGINE UR UM 103, Department of Medical Imaging, Montpellier University, Nîmes University Hospital, 30029 Nîmes, France; 2 Urology department, Nîmes University Hospital, 30029 Nîmes, France

## Introduction

Benign prostatic hyperplasia (BPH) is a highly prevalent condition, affecting approximately 42% of men over 50 and nearly 87% of men over 80 [1, 2]. It is characterized by lower urinary tract symptoms (LUTS) such as dysuria, nocturia, urgency, and frequency [3], which significantly impair patients'quality of life [4]. If left untreated, BPH can lead to complications such as obstructive renal insufficiency, vesical diverticula, and urinary lithiasis [5].

Although medical therapies targeting hormonal imbalances (e.g., 5α-reductase inhibitors and α-blockers) are commonly used, up to 42% of patients remain resistant to these treatments [6, 7]. Invasive surgical options, such as transurethral resection of the prostate (TURP) and open prostatectomy, are highly effective, but carry risks of complications, including hematuria, sexual dysfunction, and urethral strictures [8, 9]. In recent years, prostatic artery embolization (PAE) has emerged as a minimally invasive alternative, demonstrating efficacy in alleviating LUTS with fewer severe complications [10–12].

Despite its promise, approximately 20% of patients do not achieve desired clinical outcomes after PAE [13]. This failure rate represents a significant clinical challenge, as it means that a notable proportion of patients undergo an invasive procedure without experiencing the intended relief from their debilitating symptoms. This necessitates further interventions, exposing patients to additional risks and costs, and ultimately delays effective management of their condition. This variability underscores the need for better predictive tools to identify patients most likely to benefit from the procedure. While several studies have explored the clinical and anatomical predictors of PAE success, such as prostate volume and bilateral embolization [14, 15], the role of functional imaging biomarkers remains underexplored. Given the limitations of current predictive measures for PAE outcomes, we investigated the potential of DCE-MRI, a technique that offers detailed insights into tissue vascularity and permeability, to fill this gap.Dynamic contrast-enhanced MRI (DCE-MRI) perfusion parameters, such as the area under the curve (AUC) and volume transfer constant (Ktrans), have shown promise for characterizing prostatic tissue vascularity and permeability, particularly in prostate cancer [16, 17]. However, their utility in predicting PAE outcomes in BPH patients has not been thoroughly investigated. Therefore, this study aimed to assess the potential of DCE-MRI as a non-invasive tool for predicting PAE outcomes in BPH treatment and to identify specific perfusion parameters that significantly correlate with PAE success or failure.

## Materials and methods

### Study population

This retrospective study was conducted at Nimes University Hospital and included consecutive patients who underwent PAE for BPH between April 2017 and September 2020. The study was approved by the institutional review board (IRB number: 18.03.05), and the need for informed consent was waived owing to the retrospective nature of the study.

### Inclusion criteria

Patients with symptomatic BPH who underwent unilateral or bilateral PAE were included in the study. All patients had availability of pre-procedural MRI DCE-MRI perfusion data. Complete clinical follow-up data at 3 months post-PAE was also required for inclusion.

### Exclusion criteria

Patients were excluded if PAE was performed for prostatic adenocarcinoma, if baseline or follow-up clinical/imaging data was missing, if failed bilateral catheterization occurred due to anatomical variants, arterial spasm, or atheromatous occlusion, or if DCE-MRI sequences were lacking before embolization.

### Imaging procedure

All patients underwent preprocedural DCE-MRI using a 3 T MR scanner (Magnetom Verio, Siemens Healthineers, Erlangen, Germany). The imaging protocol was standardized as follows: Patients received a Normacol enema before the examination to reduce bowel motion artifacts. Glucagon (1 mg) was administered intravenously to minimize intestinal peristalsis. Gadolinium-based contrast agent (Dotarem, Guerbet, France) was injected intravenously at a dose of 0.1 mmol/kg, followed by a 20 mL saline flush. A T1-weighted gradient-echo sequence was acquired with the following parameters: TR/TE = 4.2/1.4 ms, flip angle = 12°, slice thickness = 3 mm, matrix size = 256 × 256, and temporal resolution = 5 s.

### Image interpretation and dynamic perfusion parameters

**Fig. 1:**
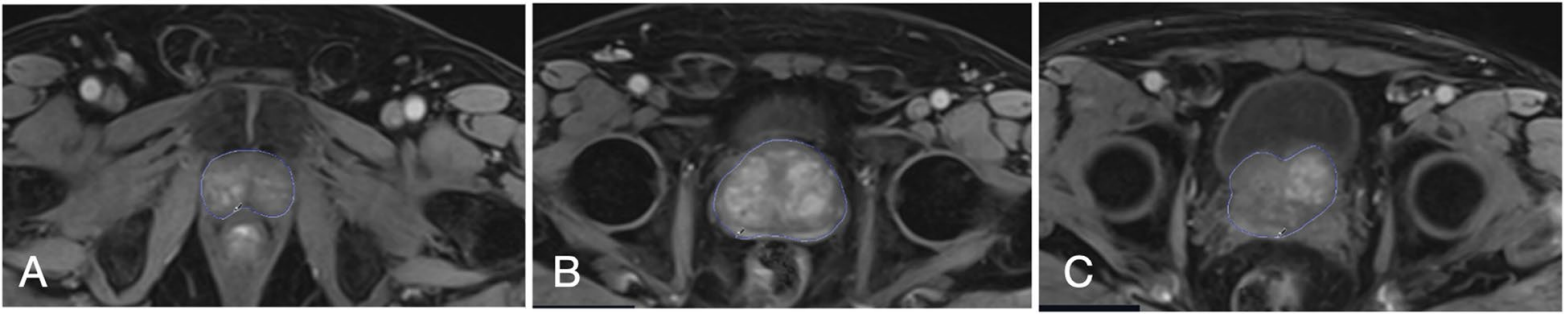
Axial T1-weighted dynamic contrast-enhanced (DCE) MRI with fat saturation following gadolinium injection, demonstrating manual delineation of the prostate at (**A**) the base, (**B**) mid-gland, and (**C**) apex

Image interpretation and dynamic perfusion parameters were analyzed using the Syngovia MR Tissue 4D software (Siemens AG, Erlangen, Germany). Multiple axial regions of interest (ROIs) were manually delineated by an experienced radiologist with > 10 years of expertise in prostate MRI (Fig. 1). To ensure consistency, the radiologist followed a detailed anatomical guideline for ROI placement. The ROIs were drawn to encompass both the peripheral and transition zones across several slices of the prostate, spanning from the base to the apex of the gland, while excluding any periprostatic tissue. Anatomical landmarks, such as the prostatic capsule and zonal anatomy, were used to guide ROI delineation. Given that the prostate extends across multiple slices, ROIs were drawn on 5 or more representative slices, ensuring coverage from the base to the apex of the gland. When the median lobe protruded into the bladder, it was included in the ROI delineation. This approach was designed to capture the overall perfusion characteristics of the prostate, considering the diffuse and heterogeneous nature of BPH. A perfusion map of the prostate was generated by processing data from these multiple ROIs, enabling a comprehensive assessment of perfusion parameters throughout the gland. This standardized protocol was applied consistently across all patients.

The following perfusion parameters were quantified: Wash-in (Win): Rate of contrast agent uptake during the initial phase; Wash-out (Wout): Rate of contrast agent clearance; Arrival Time (AT): Time for the contrast agent to first appear in the tissue; Time-to-Peak (TTP): Time taken to reach maximum contrast concentration; Peak Enhancement Intensity (PEI): Maximum signal intensity achieved post-contrast; Area Under the Curve (AUC): Integral of the concentration–time curve, reflecting total contrast agent passage through the tissue; Volume Transfer Constant (Ktrans): Rate of contrast transfer from plasma to the extracellular extravascular space (EES); Extracellular Volume (Ve): Fraction of tissue volume occupied by the EES; and Rate Constant (Kep): Rate of contrast transfer from EES back to plasma (Kep = Ktrans/Ve). To accurately quantify these parameters, kinetic calculations were performed using the"intermediate"artery input function (AIF), which represents the concentration of the contrast agent in arterial blood over time. A constant T1 value of 2000 ms was applied in these calculations to standardize the longitudinal relaxation time of the tissue, ensuring consistent and reliable measurements of tissue perfusion and permeability [18].

### Prostatic artery embolization procedure

All the PAE procedures were performed under medical sedation to ensure patient comfort. Arterial access was obtained using a 4-French introducer preferentially through the common femoral artery. In cases with challenging anatomy, the left radial artery was used as an alternative. Fluoroscopic guidance was used throughout the procedure to facilitate catheter navigation and positioning.

#### Initial imaging and planning

A pigtail catheter was positioned in the terminal aorta and cone-beam CT angiography was performed using an automatic injection of 40 mL of contrast medium at a flow rate of 15 mL/s. This step provides detailed anatomical assessment and precise planning for embolization.

#### Catheterization and embolization

**Fig. 2:**
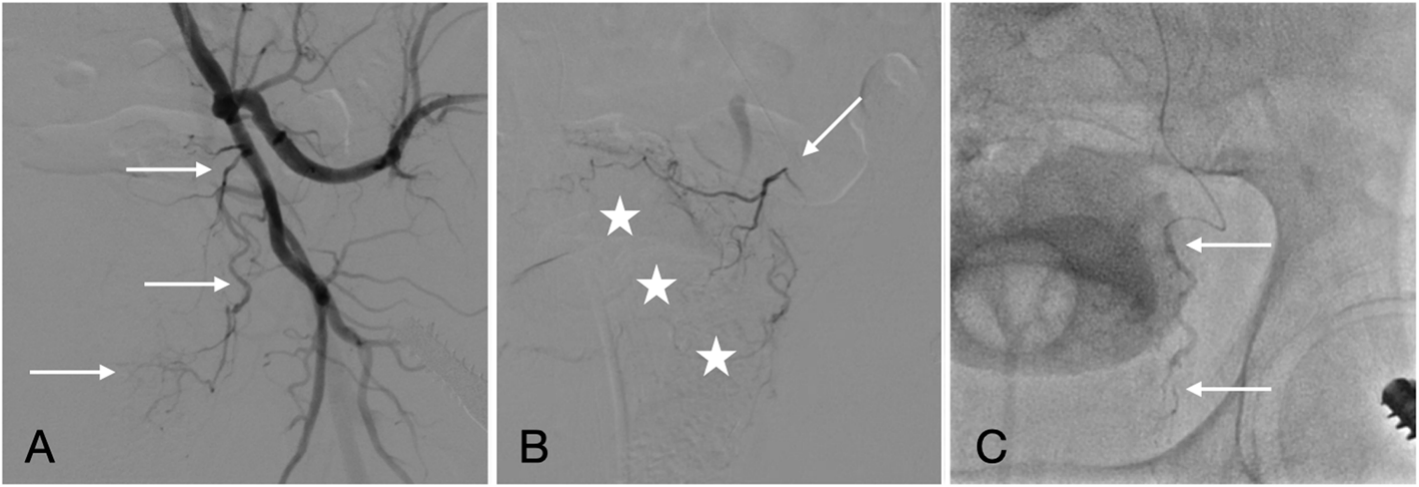
Catheterization and embolization of the prostatic artery. **A** Ipsilateral oblique projection angiogram demonstrating the origin and course of the left prostatic artery (*arrows*). **B** Selective microcatheterization of the prostatic artery (*arrow*) with injection of contrast medium, revealing the characteristic parenchymal blush of the prostate. **C** Controlled injection of 400 μm embolic particles (suspended in a contrast-saline mixture) under fluoroscopic guidance to minimize reflux and non-target embolization (arrows)

The anterior division of the contralateral internal iliac artery was accessed using an ipsilateral oblique view and a Cobra 2 catheter, with a Contra catheter employed in cases of difficult crossover. The prostatic artery was selectively catheterized using a Progreat® 2.0 French microcatheter (Terumo Medical, Tokyo, Japan). Embolization was performed using 400 μm non-spherical polyvinyl alcohol (PVA) particles (HydroPearl®, Terumo Medical). PVA particles were mixed with a contrast agent in a solution of 2 mL particles, 8 mL Visipaque 320 mg/L, and 10 mL saline. Embolization was conducted gradually to avoid reflux, with 5 mL of saline used to rinse the catheter after each 1 mL of the particle solution (Carnevale et al., 2013). The volume of injected particles was continuously monitored to ensure accurate dosing. Technical success was defined as complete flow stasis in the prostatic artery (Fig. 2).

The Cobra catheter was used to select the ipsilateral internal iliac artery via the Waltman loop technique, with a SIM 2 catheter employed as an alternative in cases of challenging anatomy. The same protocol was applied to the contralateral side, when feasible.

#### Anastomotic evaluation

**Fig. 3:**
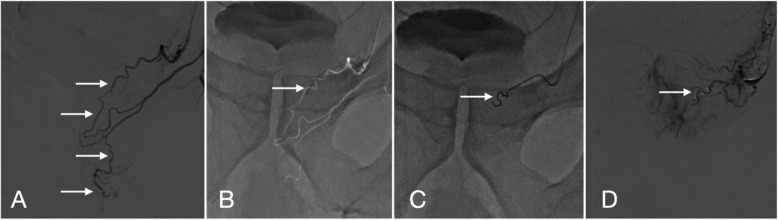
Management of pelvic-penile anastomosis during prostatic artery embolization. **A** Prostatic artery angiogram (digital subtraction angiography, DSA) via microcatheter demonstrating a pelvic-penile anastomosis (arrow), a potential pathway for non-target embolization. **B** Superselective catheterization of the anastomotic channel (arrow) under fluoroscopic guidance to isolate the prostatic vasculature. **C** Coil embolization (arrow) of the anastomosis using metallic coils to prevent collateral flow to the penis. **D** Post-embolization prostatic artery angiogram confirming successful occlusion of the anastomosis (arrow), allowing safe embolization of the prostatic artery

Throughout the procedure, the interventional radiologist carefully evaluated the anastomotic branches between the prostatic arteries and adjacent penile or rectal circulation. Selective embolization of the anastomoses was performed to prevent pelvic ischemic complications (Fig. 3).

#### Post-procedure care

Patients were typically managed under a day hospital protocol, allowing for same-day discharge after a brief observation period. However, in cases with a significant medical history or comorbidities, patients were hospitalized for 24 h for closer monitoring, in accordance with the institutional anesthesia protocol. Discharge was contingent on the absence of complications.

### Clinical outcome and measures

#### Follow-up protocol

All patients underwent a standardized follow-up protocol three months post-PAE. This follow-up protocol is the standard clinical practice at our institution. The follow-up included a clinical consultation with either a urologist or interventional radiologist, as well as a prostatic MRI. During the consultation, the following assessments were performed: International Prostate Symptom Score (IPSS): A validated questionnaire assessing the severity of lower urinary tract symptoms (LUTS). The IPSS ranges from 0 to 35, with higher scores indicating more severe symptoms. Quality of Life (QoL) Score: A single-question assessment evaluating the patient’s perceived impact of urinary symptoms on daily life. The QoL score ranged from 0 (delighted) to 6 (terrible). Bladder Catheter Status: For patients with an indwelling bladder catheter before PAE, the ability to remove the catheter was assessed.

#### Definition of clinical success

Clinical success was defined according to the Cardiovascular and Interventional Radiological Society of Europe (CIRSE) Standards of Practice on PAE. Success was defined as meeting at least one of the following criteria: A reduction in the IPSS score of ≥ 25% compared to baseline, with a follow-up IPSS score < 18. A decrease in the QoL score of ≥ 1 point compared to baseline, with a follow-up QoL score ≤ 3. Successful removal of the bladder catheter in patients who had an indwelling catheter prior to the procedure [19].

#### Definition of clinical failure

Clinical failure was defined according to the CIRSE Standards of Practice on PAE. Clinical failure was defined as any of the following: A reduction in the IPSS score of < 25% compared to baseline, or a follow-up IPSS score ≥ 18. No change or an increase in the QoL score, or a follow-up QoL score ≥ 4. Inability to remove the bladder catheter in patients who had an indwelling catheter prior to the procedure [19].

#### Patient categorization

Based on the follow-up assessments, patients were categorized into two groups: Clinical Success Group: Patients who met the criteria for clinical success; Clinical Failure Group: Patients who met the criteria for clinical failure.

#### Primary and secondary endpoints

Primary Endpoint: The difference in DCE-MRI perfusion parameters (Win, Wout, AT, TTP, PEI, AUC, Ktrans, Ve, and Kep) between the clinical success and clinical failure groups.

##### Secondary endpoints

Correlation between baseline prostate volume and clinical outcomes. Correlation between the volume of embolic particles used and clinical outcomes. Rate of procedural complications (e.g., pelvic pain, hematuria, and urinary tract infections).

### Data collection and management

All clinical and imaging data were prospectively collected and stored in a secure, anonymized database. The data included baseline characteristics (age, prostate volume, IPSS, QoL score, and catheter status), procedural details (volume of particles used and bilaterality of PAE), and follow-up outcomes (IPSS, QoL score, catheter status, and complications).

### Statistical analyses

Statistical analyses were conducted using the Statistical Analysis Systems software (v8.3, SAS Institute, Cary, NC, USA) with a conventional two-tailed α level of 0.05. To compare the success and failure groups, the Mann–Whitney test was used for quantitative variables and the Fisher exact test was used for qualitative binomial variables. Quantitative variables are presented as means with standard deviations or medians with interquartile ranges, whereas qualitative variables are reported as frequencies and corresponding proportions.

The correlation between perfusion parameters was assessed using the Pearson correlation coefficient and associated tests. Multivariate analysis was performed to identify predictive factors for successful PAE. Forward selection through logistic regression was used to build the multivariate model. This method starts with no predictors in the model and sequentially adds the most statistically significant variable at each step, provided it meets the entry criterion (*p* < 0.05). The variables considered for inclusion were age, prostate volume, volume of microspheres injected, and bilaterality of the procedure, as these factors are clinically relevant. The forward selection process continued until no remaining variable met the entry criterion. The discriminative performance of the final model was evaluated using the receiver operating characteristic (ROC) curve and the AUC [20]. The sensitivity, specificity, positive predictive value (PPV), and negative predictive value (NPV) for various thresholds were reported. The Youden index was calculated for each threshold to assess the overall performance of the model.

## Results

### Patient characteristics

**Fig. 4:**
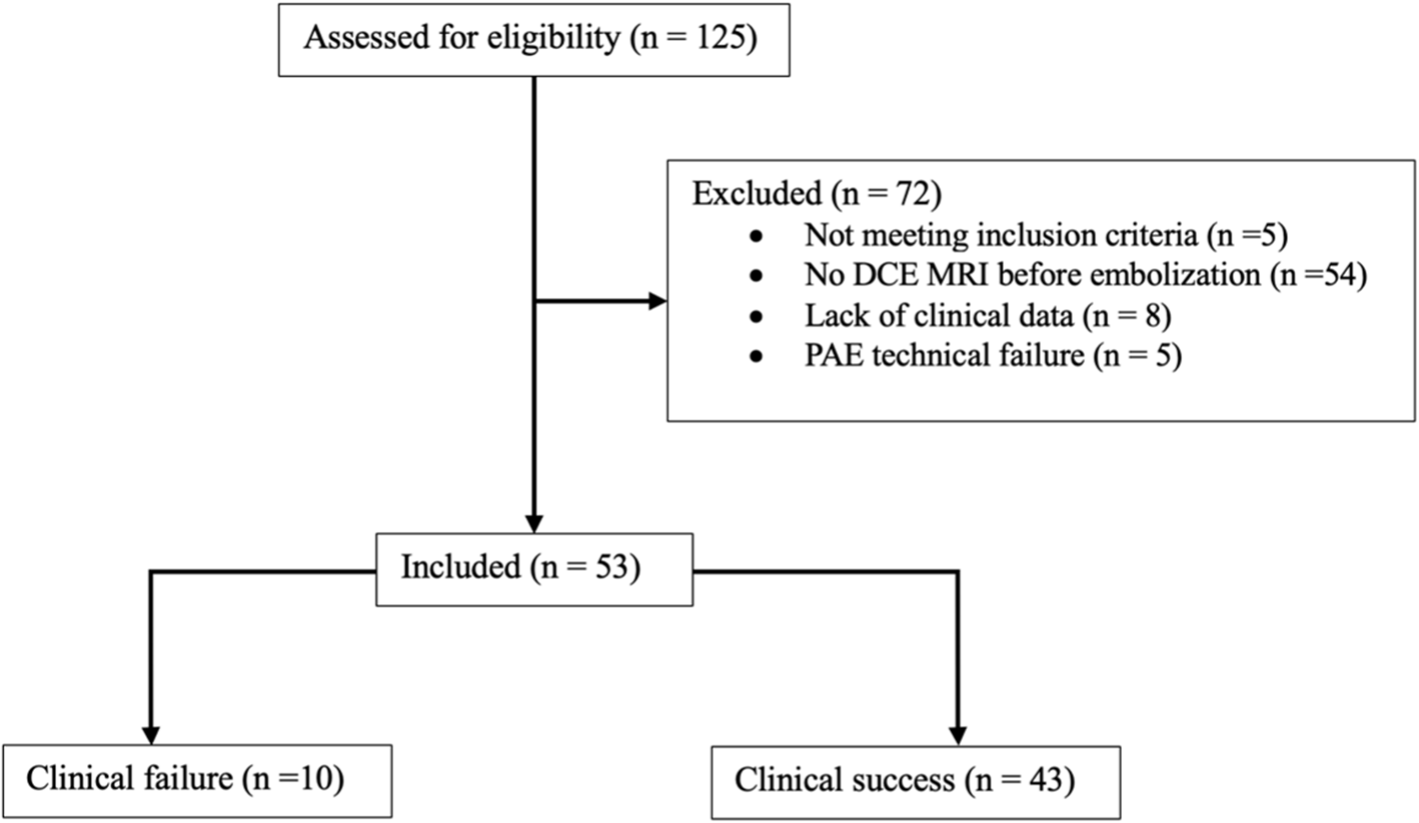
Flow chart

Between April 2017 and September 2020, a total of 125 patients underwent PAE at our institution. After applying the exclusion criteria, 53 patients were included in the final analysis (Fig. 4). The mean age of the cohort was 77 years (IQR: 67–83,25), and the mean prostate volume was 85 mL (IQR: 60–120). The baseline characteristics are summarized in Table 1.

**Table 1:** Patients'characteristics at baseline

	**Success group median (IQ range)**	**Failure group median (IQ range)**	**Total median (IQ range)**	***p*-value**
Number of patients	**43**	**10**	**53**	
Age (years)	75 (67—81)	82 (78—86)	77 (67—83,25)	**0,05**
IPSS before embolization	19 (13–23)	24 (20–26)	19 (14–25)	0.08
QoL before embolization	5 (4—6)	5 (4—6)	5 (4—6)	0.28
Nb of patients with pre-embolization urinary catheters	10	5	15	0,15
Prostate volume (mL)	87 (71—115)	92 (56—161)	85 (60—120)	0,87

*IQR* Interquartile range, *IPSS* International Prostate Symptom Score, *QoL* Quality of life

### Procedural outcomes

Bilateral PAE was successfully performed in 42/53 (79.2%) patients. Unilateral PAE was performed in of 11/53 (20.7%) patients because of technical challenges, including failed catheterization (*n* = 5), absence of a contralateral prostatic artery (*n* = 4), vasospasm (*n* = 1), and atheromatous occlusion (*n* = 1). The mean volume of PVA particles used was 10.8 mL (range: 3–26 mL). Technical procedure data are detailed in Tables 2 and 3

**Table 2:** Technical procedure data

**Variables** Median (IQR: Q1-Q3)	**Success group** (*n* = 43)	**Failure group** (*n* = 10)	**Total** (*n* = 53)	***p*-value**
**Injected particle volume** (mL)	9 (6–13)	6 (5–7)	9 (6–13)	***0.01***
**Initial prostate volume/particle volume** (mL)	8.8 (6.8–13.4)	10.6 (9.4–19.0)	8.5 (6.8–13.4)	0.17
**Unilateral procedures**, n (%)	9 (20.9)	2 (20)	11 (20.7)	0.69
**DSP** (Gy.cm^2^)	252 390 (184.583–304.357)	216 870 (152.913–284.745)	252 389 (166.760–300.685)	0.53
**Fluoroscopy time** (min)	46 (34–52)	39 (28–48)	44 (32–53)	0.11

*IQR* Interquartile range, *DSP* Dose surface product

**Table 3:** Prostate MRI perfusion parameters

Perfusion parameter	Success group median (IQR)	Failure group median (IQR)	Total median (IQR)	*p*-value
Win	0,22 (0,19—0,28)	0,17 (0,13—0,19)	0,17 (0,13—0,19)	**0.001**
Wout	0,08 (0,06—0,08)	0,04 (0,03—0,04)	0,04 (0,03—0,06)	**0.001**
AT	0,61 (0,55—0,74)	0,74 (0,61—0,81)	0,74 (0,61—0,81)	0.12
TTP	0,54 (0,53—0,60)	0,68 (0,54—0,69)	0,68 (0,54—0,69)	**0.04**
PEI	0,19 (0,16—0,22)	0,15 (0,10—0,18)	0,15 (0,10—0,18)	**0.016**
AUC	0,11 (0,9 −0,13)	0,9 (0,7—0,10)	0,7 (0,7—0,10)	**0.04**
Ktrans	0,09 (0,07—0,11)	0,6 (0,4—0,7)	0,06 (0,04—0,07)	**0.014**
Ve	0,30 (0,23—0,49)	0,32 (0,19—0,43)	0,32 (0,19—0,43)	0.56
Kep	0,27 (0,17—0,41)	0,25 (0,15—0,29)	0,25 (0,15—0,29)	0.46

*Win* Wash-in, *Wout* Wash-out, *AT* Arrival time, *TTP* Time-to-peak, *PEI* Peak enhancement intensity, *AUC* Area under the curve, *Ktrans* Volume transfer constant, *Ve* Extracellular volume, *Kep* Rate constant

### Clinical outcomes

At the 3-month follow-up, clinical success was achieved in of 44/53 (83%) patients. The remaining 9/53 (17%) patients were classified as having clinical failures. In the clinical success group, the median IPSS score decreased from 22 (IQR: 18–26) at baseline to 10 (IQR: 7–14) at follow-up (*p* < 0.001). The median QoL score improved from five (IQR: 4–5) to two (IQR: 1–3) (*p* < 0.001). Among patients with an indwelling catheter at baseline (*n* = 12), successful catheter removal was achieved in 10/12 (83.3%) cases.

### Univariate analysis

Univariate analysis identified several factors associated with clinical success. Patients in the clinical success group were significantly younger (median age: 74 years, IQR: 67–81) compared to the clinical failure group (median age: 81 years, IQR: 78–87) (*p* = 0.02). The clinical success group had significantly higher median AUC (0.112, IQR: 0.092–0.134) and Ktrans (0.085, IQR: 0.066–0.108) compared to the clinical failure group (AUC: 0.09, IQR: 0.06–0.098; Ktrans: 0.064, IQR: 0.043–0.077) (*p* < 0.01 for both). The median volume of particles used was higher in the clinical success group (9 mL, IQR: 6–13) compared to the clinical failure group (5.5 mL, IQR: 5–7) (*p* = 0.01). The results of the univariate analysis are presented in Table 4.

**Table 4:** Univariate analysis of clinical, procedural, and perfusion parameters

		**Failure**	**Success**		
**variable**	**modalities**	**n (%)**	**n (%)**	**Test**	**p-value**
**Bilateral embolization**	*No*	2 (20)	9 (20.9)	F	0.69
	*Yes*	8 (80)	34 (79.07)		
**Age**	*Mean (*± *SD)*	81.82 (± 7.87)	74.11 (± 10.10)	MW	**0.02**
	*Median (Q25;Q75)*	81.00 (78.00; 87.00)	74.50 (67.00; 81.00)		
	*N*	10	43		
**PrV**	*Mean (*± *SD)*	101.07 (± 64.81)	96.71 (± 47.90)	MW	0.83
	*Median (Q25;Q75)*	65.00 (50.77; 167.69)	85.58 (66.21; 116.32)		
	*N*	10	43		
**Ktrans**	*Mean (*± *SD)*	0.060 (± 0.021)	0.090 (± 0.039)	MW	** < 0.01**
	*Median (Q25;Q75)*	0.064 (0.043; 0.077)	0.085 (0.066; 0.108)		
	*N*	10	43		
**AUC**	*Mean (*± *SD)*	0.080 (± 0.027)	0.113 (± 0.039)	MW	** < 0.01**
	*Median (Q25;Q75)*	0.090 (0.060; 0.098)	0.112 (0.092; 0.134)		
	*N*	10	43		
**IPV**	*Mean (*± *SD)*	6.80 (± 3.77)	11.02 (± 6.40)	MW	**0.01**
	*Median (Q25;Q75)*	5.50 (5.00; 7.00)	9.00 (6.00; 13.00)		
	*N*	10	43		
**PrV/IPV**	*Mean (*± *SD)*	13.93 (± 8.15)	10.10 (± 4.53)	MW	0.17
	*Median (Q25;Q75)*	10.64 (9.18; 20.96)	8.81 (6.81; 13.38)		
	*N*	10	43		

*IPV* Injected particle volume, *PrV* Prostate volume, *F* Fisher test, *WMW* Mann–Whitney test

### Perfusion multivariate analysis

To identify independent predictors of clinical success, a multivariate logistic regression analysis was performed using forward selection, adjusting for potential confounders including age, baseline prostate volume, volume of embolic particles injected, and bilaterality of the PAE procedure. The analysis identified two significant independent predictors of clinical success: Younger Age (OR = 0.877; 95% CI: 0.792–0.972; *p* = 0.010) and higher baseline AUC from DCE-MRI (OR = 1.406; 95% CI: 1.092–1.810; *p* = 0.010). This indicates that for every one-year increase in patient age, the odds of achieving clinical success decreased by approximately 12.3%. Conversely, for every unit increase in the baseline AUC value, the odds of clinical success increased by approximately 40.6%.

### Correlations

Significant positive correlations were observed between baseline prostate volume and the volume of embolic particles used (ρ = 0.555, *p* < 0.0001), between Ktrans and AUC (ρ = 0.69, *p* < 0.0001), and between the volume of embolic particles used and AUC (ρ = 0.33, *p* = 0.015). Correlation coefficients are detailed in Table 5."

**Table 5:** Correlation between quantitative parameters

Pearson correlation coefficients					
	**AUC**	**Ktrans**	**Age**	**IPV**	**PrV**
**AUC**	1.00000 53	0.69323 ** <.0001** 53	0.05955 0.6658 53	0.33296 **0.0148** 53	0.07877 0.5713 53
**Ktrans**		1.00000 53	0.17505 0.2011 53	0.29062 **0.0348** 53	0.05527 0.6914 53
**Age**			1.00000 53	0.02215 0.8749 53	0.19442 0.1589 53
**IPV**				1.00000 53	0.55549 ** <.0001** 53
**PrV**					1.00000 53

*IPV* Injected particle volume, *PrV* Prostate volume

## Discussion

Prostatic artery embolization represents a significant advance in minimally invasive treatment for LUTS secondary to BPH [10–12]. However, optimizing patient selection remains crucial, given that a subset of patients do not experience the desired clinical improvement [13]. This study explored whether pre-procedural DCE-MRI perfusion parameters could enhance prediction of early PAE outcomes. Our findings indicate that two factors – younger patient age and higher baseline AUC from DCE-MRI – independently predicted clinical success at the 3-month follow-up point.

The emergence of AUC as an independent predictor is noteworthy. It suggests that functional information about prostate vascularity, captured by DCE-MRI, provides prognostic value beyond established predictors like prostate volume or achieving bilateral embolization [14, 15]. Although baseline prostate volume was not significantly different between our success and failure groups, the association between higher AUC and success implies that the tissue's perfusion characteristics may significantly influence treatment response. Plausibly, higher baseline perfusion could facilitate more effective embolic agent delivery and subsequent ischemia within the target tissue. This interpretation is supported by the observed correlation between AUC and Ktrans (*p* < 0.001), another parameter reflecting tissue perfusion and permeability [18].

Regarding patient age, our finding that younger age predicted better early outcomes is consistent with some previous PAE studies [13], although the literature is not entirely uniform on this point [14]. Such discrepancies might arise from variations in study populations, success criteria, or follow-up durations. Nevertheless, the independent predictive contribution of both age and AUC in our multivariate model (*p* = 0.010 for both) highlights the potential benefit of integrating clinical data with functional imaging for improved patient stratification and managing expectations regarding early treatment response.

However, the clinical implementation of AUC as a predictive biomarker faces practical challenges. A primary concern is the known variability in quantitative DCE-MRI measurements across different MRI systems, acquisition protocols, and analysis platforms [18]. This variability complicates the establishment of universal AUC thresholds. Consequently, while our findings within our institutional setting are encouraging, the specific AUC values identified may not be directly transferable to other centers. Widespread clinical adoption would likely require significant efforts toward protocol standardization or the development and validation of normalized or relative perfusion metrics less susceptible to inter-scanner variability.

Several limitations inherent to this study warrant consideration. Its retrospective nature introduces potential for selection bias and unmeasured confounding. The relatively small sample size (*n* = 53), particularly the limited number in the clinical failure group (*n* = 9), restricts the statistical power and necessitates caution regarding the generalizability of the findings; validation in larger cohorts is essential. As a single-center study, results may be influenced by local demographics and practices. Furthermore, and critically, the 3-month follow-up assesses only early outcomes. It does not address the long-term durability of symptom relief, a key factor for clinical decision-making. Whether these early predictors correlate with sustained benefit remains an open question. Finally, manual ROI delineation, although performed by an experienced radiologist, carries potential for inter-observer variability.

Future research should prioritize addressing these limitations. Prospective, multi-center trials are needed to validate these findings using standardized DCE-MRI protocols, which would also help assess the impact of inter-institutional variability. Such studies should incorporate robust methods for perfusion analysis, potentially including automated techniques leveraging machine learning, which have shown promise in other prostate MRI applications [21], to enhance reproducibility. Crucially, extending the follow-up duration to at least one to two years in future trials is essential to evaluate whether baseline parameters like AUC predict not just early response but also the long-term durability of PAE outcomes.

In conclusion, this study provides evidence that younger age and higher baseline prostate perfusion (AUC) measured by DCE-MRI are independent predictors of early clinical success following PAE. This highlights the potential of functional imaging to contribute to patient selection. Nonetheless, owing to significant limitations – including the retrospective design, small sample size, short-term follow-up, and the technical challenges of standardizing DCE-MRI – these findings should be considered preliminary. Further validation through large-scale, prospective studies with standardized methods and long-term follow-up is imperative before baseline perfusion parameters can be reliably incorporated into clinical practice for guiding PAE decisions.

## Data Availability

The datasets generated during the current study are not publicly available due to patient confidentiality restrictions but are available from the corresponding author on reasonable request.

## References

[1] Guess HA, Arrighi HM, Metter EJ, Fozard JL. Cumulative prevalence of prostatism matches the autopsy prevalence of benign prostatic hyperplasia. Prostate. 1990;17(3):241–6. 1700403 10.1002/pros.2990170308

[2] Berry SJ, Coffey DS, Walsh PC, Ewing LL. The development of human benign prostatic hyperplasia with age. J Urol. 1984;132(3):474–9. 6206240 10.1016/s0022-5347(17)49698-4

[3] Mobley D, Feibus A, Baum N. Benign prostatic hyperplasia and urinary symptoms: Evaluation and treatment. Postgrad Med. 2015;127(3):301–7. 25823641 10.1080/00325481.2015.1018799

[4] Barry MJ. Medical outcomes research and benign prostatic hyperplasia. Prostate Suppl. 1990;3:61–74. 1689171 10.1002/pros.2990170507

[5] Olbrich O, Woodford-Williams E, Irvine RE, Webster D. Renal function in prostatism. Lancet. 1957;272(6983):1322–4. 13440039 10.1016/s0140-6736(57)91845-7

[6] Brown CT, Yap T, Cromwell DA, Rixon L, Steed L, Mulligan K, et al. Self management for men with lower urinary tract symptoms: randomised controlled trial. BMJ. 2007;334(7583):25. 17118949 10.1136/bmj.39010.551319.AE PMC1764065

[7] Roehrborn CG, Siami P, Barkin J, Damião R, Major-Walker K, Nandy I, et al. The effects of combination therapy with dutasteride and tamsulosin on clinical outcomes in men with symptomatic benign prostatic hyperplasia: 4-year results from the CombAT study. Eur Urol. 2010;57(1):123–31. 19825505 10.1016/j.eururo.2009.09.035

[8] Ahyai SA, Gilling P, Kaplan SA, Kuntz RM, Madersbacher S, Montorsi F, et al. Meta-analysis of functional outcomes and complications following transurethral procedures for lower urinary tract symptoms resulting from benign prostatic enlargement. Eur Urol. 2010;58(3):384–97. 20825758 10.1016/j.eururo.2010.06.005

[9] Oelke M, Bachmann A, Descazeaud A, Emberton M, Gravas S, Michel MC, et al. EAU guidelines on the treatment and follow-up of non-neurogenic male lower urinary tract symptoms including benign prostatic obstruction. Eur Urol. 2013;64(1):118–40. 23541338 10.1016/j.eururo.2013.03.004

[10] Pisco JM, Bilhim T, Pinheiro LC, Fernandes L, Pereira J, Costa NV, et al. Medium- and Long-Term Outcome of Prostate Artery Embolization for Patients with Benign Prostatic Hyperplasia: Results in 630 Patients. J Vasc Interv Radiol. 2016;27(8):1115–22. 27321890 10.1016/j.jvir.2016.04.001

[11] Uflacker A, Haskal ZJ, Bilhim T, Patrie J, Huber T, Pisco JM. Meta-Analysis of Prostatic Artery Embolization for Benign Prostatic Hyperplasia. J Vasc Interv Radiol. 2016;27(11):1686-1697.e8. 27742235 10.1016/j.jvir.2016.08.004

[12] Sapoval M, Thiounn N, Descazeaud A, Déan C, Ruffion A, Pagnoux G, et al. Prostatic artery embolisation versus medical treatment in patients with benign prostatic hyperplasia (PARTEM): a randomised, multicentre, open-label, phase 3, superiority trial. Lancet Reg Health Eur. 2023;31: 100672. 37415648 10.1016/j.lanepe.2023.100672 PMC10320610

[13] Maclean D, Harris M, Drake T, Maher B, Modi S, Dyer J, et al. Factors Predicting a Good Symptomatic Outcome After Prostate Artery Embolisation (PAE). Cardiovasc Intervent Radiol. 2018;41(8):1152–9. 29484467 10.1007/s00270-018-1912-5

[14] Bilhim T, Pisco J, Pereira JA, Costa NV, Fernandes L, Campos Pinheiro L, et al. Predictors of Clinical Outcome after Prostate Artery Embolization with Spherical and Nonspherical Polyvinyl Alcohol Particles in Patients with Benign Prostatic Hyperplasia. Radiology. 2016;281(1):289–300. 27223621 10.1148/radiol.2016152292

[15] Hacking N, Vigneswaran G, Maclean D, Modi S, Dyer J, Harris M, et al. Technical and Imaging Outcomes from the UK Registry of Prostate Artery Embolization (UK-ROPE) Study: Focusing on Predictors of Clinical Success. Cardiovasc Intervent Radiol. 2019;42(5):666–76. 30603967 10.1007/s00270-018-02156-8

[16] Cindil E, Oner Y, Sendur HN, Ozdemir H, Gazel E, Tunc L, et al. The Utility of Diffusion-Weighted Imaging and Perfusion Magnetic Resonance Imaging Parameters for Detecting Clinically Significant Prostate Cancer. Can Assoc Radiol J. 2019;70(4):441–51. 31561925 10.1016/j.carj.2019.07.005

[17] Hoang Dinh A, Melodelima C, Souchon R, Lehaire J, Bratan F, Mège-Lechevallier F, et al. Quantitative Analysis of Prostate Multiparametric MR Images for Detection of Aggressive Prostate Cancer in the Peripheral Zone: A Multiple Imager Study. Radiology. 2016;280(1):117–27. 26859255 10.1148/radiol.2016151406

[18] Tofts PS, Brix G, Buckley DL, Evelhoch JL, Henderson E, Knopp MV, et al. Estimating kinetic parameters from dynamic contrast-enhanced T(1)-weighted MRI of a diffusable tracer: standardized quantities and symbols. J Magn Reson Imaging. 1999;10(3):223–32. 10508281 10.1002/(sici)1522-2586(199909)10:3<223::aid-jmri2>3.0.co;2-s

[19] Cornelis FH, Bilhim T, Hacking N, Sapoval M, Tapping CR, Carnevale FC. CIRSE Standards of Practice on Prostatic Artery Embolisation. Cardiovasc Intervent Radiol. 2020;43(2):176–85. 31792588 10.1007/s00270-019-02379-3

[20] Hanley JA, McNeil BJ. The meaning and use of the area under a receiver operating characteristic (ROC) curve. Radiology. 1982;143(1):29–36. 7063747 10.1148/radiology.143.1.7063747

[21] Cuocolo R, Cipullo MB, Stanzione A, Ugga L, Romeo V, Radice L, et al. Machine learning applications in prostate cancer magnetic resonance imaging. Eur Radiol Exp. 2019;3(1):35. 31392526 10.1186/s41747-019-0109-2 PMC6686027

